# Contrasting transcriptional responses of PYR1/PYL/RCAR ABA receptors to ABA or dehydration stress between maize seedling leaves and roots

**DOI:** 10.1186/s12870-016-0764-x

**Published:** 2016-04-21

**Authors:** Wenqiang Fan, Mengyao Zhao, Suxin Li, Xue Bai, Jia Li, Haowei Meng, Zixin Mu

**Affiliations:** College of Life Sciences, Northwest A&F University, Yangling, 712100 Shaanxi China

**Keywords:** ABA signal transduction pathway, Drought stress, Gene expression, ZmPYLs, ZmPP2C, ZmSnRK2

## Abstract

**Background:**

The different actions of abscisic acid (ABA) in the aboveground and belowground parts of plants suggest the existence of a distinct perception mechanism between these organs. Although characterization of the soluble ABA receptors PYR1/PYL/RCAR as well as core signaling components has greatly advanced our understanding of ABA perception, signal transduction, and responses, the environment-dependent organ-specific sensitivity of plants to ABA is less well understood.

**Results:**

By performing real-time quantitative PCR assays, we comprehensively compared transcriptional differences of core ABA signaling components in response to ABA or osmotic/dehydration stress between maize (*Zea mays* L.) roots and leaves. Our results demonstrated up-regulation of the transcript levels of *ZmPYL*s homologous to dimeric-type *Arabidopsis* ABA receptors by ABA in maize primary roots, whereas those of *ZmPYL*s homologous to monomeric-type *Arabidopsis* ABA receptors were down-regulated. However, this trend was reversed in the leaves of plants treated with ABA via the root medium. Although the mRNA levels of *ZmPYL1-3* increased significantly in roots subjected to polyethylene glycol (PEG)-induced osmotic stress, *ZmPYL4-11* transcripts were either maintained at a stable level or increased only slightly. In detached leaves subjected to dehydration, the transcripts of *ZmPYL1-3* together with *ZmPYL5, ZmPYL6, ZmPYL10* and *ZmPYL11* were decreased, whereas those of *ZmPYL4, ZmPYL7* and *ZmPYL8* were significantly increased. Our results also showed that all of the evaluated transcripts of PP2Cs and SnRK2 were quickly up-regulated in roots by ABA or osmotic stress; conversely they were either up-regulated or maintained at a constant level in leaves, depending on the isoforms within each family.

**Conclusions:**

There is a distinct profile of PYR/PYL/RCAR ABA receptor gene expression between maize roots and leaves, suggesting that monomeric-type ABA receptors are mainly involved in the transmission of ABA signals in roots but that dimeric-type ABA receptors primarily carry out this function in leaves. Given that *ZmPYL1* and *ZmPYL4* exhibit similar transcript abundance under normal conditions, our findings may represent a novel mechanism for species-specific regulation of PYR/PYL/RCAR ABA receptor gene expression. A difference in the preference for core signaling components in the presence of exogenous ABA versus stress-induced endogenous ABA was observed in both leaves and roots. It appears that core ABA signaling components perform their osmotic/dehydration stress response functions in a stress intensity-, duration-, species-, organ-, and isoform-specific manner, leading to plasticity in response to adverse conditions and, thus, acclimation to life on land. These results deepen our understanding of the diverse biological effects of ABA between plant leaves and roots in response to abiotic stress at the stimulus-perception level.

**Electronic supplementary material:**

The online version of this article (doi:10.1186/s12870-016-0764-x) contains supplementary material, which is available to authorized users.

## Background

A universally conserved adaptation to drought stress observed in plants is an adjustment of the biosynthesis and metabolism of various phytohormones [1]. Abscisic acid (ABA) is the most important hormone involved in the resistance of plants to drought and other abiotic stresses [2]. Due to the potential applications of ABA for improving the stress tolerance of cultivated plants in the field, the mechanisms underlying ABA signal transduction, especially ABA perception, have been studied extensively for the past two decades [3]. Although chloroplast membrane-localized Mg-chelatase H subunit (CHLH)/putative ABA receptor (ABAR) [4] and plasma membrane-localized GPCR-type G proteins (GTG1/2) [5] were previously reported to be ABA receptors, it has remained unclear how they modulate plant responses to ABA. A breakthrough occurred in 2009, when at least two independent groups identified and characterized the pyrabactin resistance 1(PYR1)/PYR1-like (PYL)/regulatory components of ABA receptors (RCAR) protein family as soluble ABA receptors [6–9]. Since then, a new model for ABA action has been proposed and validated. In this model, PYR/PYL/RCAR ABA receptors function at the apex of a negative regulatory pathway to directly regulate group A type 2C protein phosphatases (PP2Cs), which in turn directly regulate subclass III plant-specific sucrose nonfermenting 1-related subfamily 2 (SnRK2) protein kinases [2, 10–12]. These three effector families constitute the core components of the signaling pathway, and their members have been shown to mediate several ABA-controlled plant physiological processes, such as seed germination and dormancy, fruit maturation, seedling growth, stomatal movement and stress-related gene expression [13–19]. Moreover, these core signaling components are well conserved among higher plants, indicating that the establishment of the core ABA signaling pathway had a great impact on the colonization of land, especially with regard to drought tolerance [11, 20–22].

Research on ABA signal transduction has flourished since the identification and characterization of the core signaling components. However, many such investigations have been focused on aboveground tissues, whereas the function of ABA in root-related processes is poorly understood. It is well known that ABA has a distinct effect on aboveground leaves and belowground roots, especially under drought conditions, whereby ABA inhibits shoot growth and water release while enhancing root growth and water uptake [23, 24]. There is obvious redundancy in the modulation of seed germination, stomatal aperture and transcriptional responses to ABA in vegetative tissues by PYR1/PYL/RCAR ABA receptor genes [7, 25]. Antoni et al. [24] recently found that the single knockout of pyl8 resulted in reduced sensitivity to the ABA-mediated inhibition of root growth, and Zhao et al. [26] further demonstrated that PYL8 promotes lateral root growth independent of the core ABA-SnRK2 signaling pathway. These genetic results suggest the existence of different ABA functions corresponding to specific signaling mechanisms or that distinct preferences for components of the PYR1/PYL/RCAR signaling pathway exist between leaves and roots.

In addition to the organ specificity of ABA, it has been shown that ABA signaling is related to different types of stress, such as dehydration vs. cold stress [27], as well as the duration of stress experienced by plants [28] and plant water conditions [29, 30]. Nonetheless, the environment-dependent organ-specific sensitivity of the core signaling components remains poorly understood. Based on previous work characterizing the maize effectors ZmPYL [21, 31], ZmPP2Cs [32, 33] and ZmSnRK2s [34–37], in the present study, contrasting transcriptional responses of ABA core signaling components to ABA, PEG (osmotic stress) or dehydration stress were studied in a time-course analysis in both roots and leaves. Our objective was to explore the relationship between ABA functions and signal transduction, with a particular emphasis on the plasticity of the PYR1/PYL/RCAR-PP2C-SnRK2 signaling pathway in response to various abiotic stresses and the stress intensities that fine-tune the actions of ABA in various organs.

## Results

### Sequence analysis and alignment of core ABA signaling component genes between *Arabidopsis* and maize

Eleven ZmPYL cDNAs [21], 10 ZmSnRK2 cDNAs [34], and five ZmPP2C cDNAs identified in the present work as encoded by the maize genome exhibit great similarity to the 14, nine and 10 corresponding sequences from *Arabidopsis* (Table 1). Additional file 1: Figure S1 shows the sequence similarity determined through comparisons of functional residues and domains between *Arabidopsis* and maize proteins using CLUSTALX 2.1. As indicated in Table 1, maize genes *GRMZM2G134731* (designated *ZmPYL1*) and *AC194914.3FG002* (designated *ZmPYL2*) show identical homology to *AtPYL1* of *Arabidopsis*. Similarly, *GRMZM2G057959* and *GRMZM2G144224* share close homology with *AtPYL5*; these proteins were designated *ZmPYL5* and *ZmPYL6*, respectively. *GRMZM2G154987*, *GRMZM2G047677*, *GRMZM2G141382*, *GRMZM2G165567*, *GRMZM2G133631*, *GRMZM2G063882* and *GRMZM2G048733* display homology to *AtPYL2*, *AtPYL4*, *AtPYL6*, *AtPYL8*, *AtPYL10*, *AtPYL7*, and *AtPYL9,* respectively; the maize proteins were named *ZmPYL3, ZmPYL4, ZmPYL7, ZmPYL8, ZmPYL9*, *ZmPYL10* and *ZmPYL11*, as described in Table 1.

**Table 1:** Comparison of *PYR/PYL/RCAR*, clade-A *PP2C*, and subclass III *SnRK2* genes between *Arabidopsis thaliana* and *Zea mays* L

*Arabidopsis thaliana*			Z*ea mays* L*.*		
Gene	Locus	Length (aa)	Gene	Locus	Length (aa)
*AtPYL1*	AT5G46790	221	*ZmPYL1*	GRMZM2G134731	205
			*ZmPYL2*	AC194914.3_FG002	212
*AtPYL2*	AT2G26040	190	*ZmPYL3*	GRMZM2G154987	188
*AtPYL4*	AT2G38310	207	*ZmPYL4*	GRMZM2G047677	200
*AtPYL5*	AT5G05440	203	*ZmPYL5*	GRMZM2G057959	218
			*ZmPYL6*	GRMZM2G144224	220
*AtPYL6*	AT2G40330	215	*ZmPYL7*	GRMZM2G141382	253
*AtPYL8*	AT5G53160	188	*ZmPYL8*	GRMZM2G165567	169
*AtPYL10*	AT4G27920	183	*ZmPYL9*	GRMZM2G133631	197
*AtPYL7*	AT4G01026	211	*ZmPYL10*	GRMZM2G063882	212
*AtPYL9*	AT1G01360	187	*ZmPYL11*	GRMZM2G048733	217
*AtHAB1*	AT1G72770	406	*ZmHAB1*	BT017295	368
*AtHAB2*	AT1G17550	511			
*AtAHG1*	AT5G51760	416	*ZmPP2CA*	GRMZM2G059453	408
*AtPP2CA*	AT3G11410	399			
*AtHAI1*	AT5G59220	413	*ZmHAI1*	BT084605	394
*AtHAI2*	AT1G07430	442			
*AtHAI3*	AT2G29380	362			
*AtABI1*	AT4G26080	434	*ZmABI1*	GRMZM2G300125	394
*AtABI2*	AT5G57050	423	*ZmABI2*	GRMZM2G383807	423
*AtSnRK2.1*	AT5G08590	353	*ZmSnRK2.1*	GRMZM2G035809	342
*AtSnRK2.2*	AT3G50500	362	*ZmSnRK2.2*	GRMZM2G056732	339
*AtSnRK2.3*	AT5G66880	361	*ZmSnRK2.3*	GRMZM2G180916	333
*AtSnRK2.4*	AT1G10940	363	*ZmSnRK2.4*	GRMZM2G110922	361
*AtSnRK2.5*	AT5G63650	360	*ZmSnRK2.5*	GRMZM2G110908	363
*AtSnRK2.6*	AT4G33950	362	*ZmSnRK2.6*	GRMZM2G130018	364
*AtSnRK2.7*	AT4G40010	350	*ZmSnRK2.7*	GRMZM2G155593	356
*AtSnRK2.8*	AT1G78290	343	*ZmSnRK2.8*	GRMZM2G138861	359
*AtSnRK2.9*	AT2G23030	339			
*AtSnRK2.10*	AT1G60940	361	*ZmSnRK2.10*	GRMZM2G066867	362
			*ZmSnRK2.11*	GRMZM2G063961	359

Genes encoding ABA receptors of *Arabidopsis thaliana* were used as query to identify the orthologous proteins from *Zea mays* L. Amino acid (aa) Length and gene locus are listed

The maize genes *EU97133*6 *(GRMZM2G300125*) and *EU966462 (GRMZM2G383807)*, identified by Alexandrov et al. [38], were described as ABA-insensitive (ABI) subfamily *PP2Cs* and designated *ZmABI1* and *ZmABI2*, respectively. Similarly, *ZmPP2CA (GRMZM2G059453)*, *ZmHAB1* (locus *BT017295*) and *ZmHAI1* (locus *BT084605*) were identified in the present work, representing the best hits against *Arabidopsis* sequences, according to *tblastn* results (Table 1). Table 1 also shows sequence similarity comparisons of the *Arabidopsis* and maize SnRK2 family proteins, which were identified by Soderlund et al. [39].

### Phylogenetic analysis

**Fig. 1:**
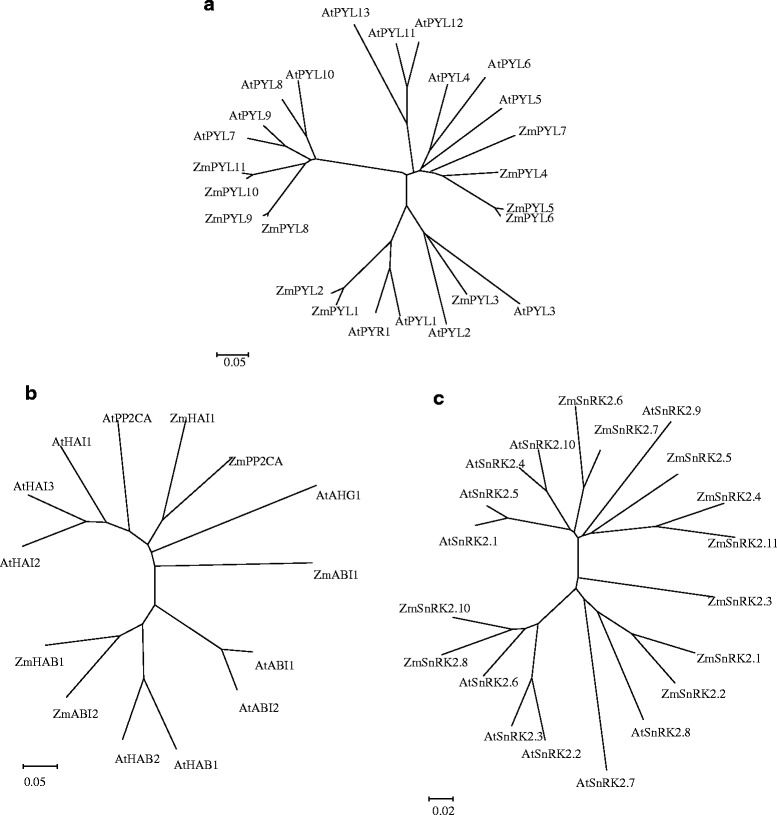
Phylogenetic relationships of the PYR/PYL/RCAR ABA receptor family (**a**), PP2C clade A (**b**) and SnRK2 family (**c**) between *Arabidopsis* and maize. An unrooted tree was drawn according to inference based on a neighbor-joining (NJ) analysis using the MEGA 5.1 program. *Arabidopsis* amino acid sequences were collected from the NCBI database. Maize sequences were obtained from *blastn* searches against *Arabidopsis* gene sets

Homology analysis revealed that the ABA core signaling components of maize are highly correlated with those of the model plant *Arabidopsis*. Phylogenetic tree and motif analyses classified the ZmPYLs into three subfamilies, comparable to those in *Arabidopsis*, with ZmPYL1, ZmPYL2 and ZmPYL3 sharing the same branch as AtPYR1, AtPYL1, AtPYL2 and AtPYL3. ZmPYL4, ZmPYL5, ZmPYL6 and ZmPYL7 grouped with the AtPYL4, AtPYL5, AtPYL6, AtPYL11, AtPYL12 and AtPYL13 branch. Moreover, ZmPYL8, ZmPYL9, ZmPYL10 and ZmPYL11 exhibit close similarity to AtPYL7, AtPYL8, AtPYL9 and ZmPYL10 (Fig. 1a).

The PP2Cs were distributed between two separate branches, with ZmHAI1 and ZmHAB1 sharing the same branches as ZmPP2CA and ZmABI2, respectively (Fig. 1b). The kinase family members ZmSnRK2.1 and ZmSnRK2.2, ZmSnRK2.6 and ZmSnRK2.7, ZmSnRK2.8 and ZmSnRK2.10 and ZmSnRK2.4, ZmSnRK2.5 and ZmSnRK2.11 clustered into the same branches (Fig. 1c). Interestingly, ZmSnRK2.3 remained independent. These results indicated that the ZmPYR/PYL/RCAR receptor, ZmPP2C, and ZmSnRK2 genes of maize correlate well with the corresponding genes in *Arabidopsis*.

### Expression of ZmPYR/PYL/RCAR mRNAs in hydroponically grown maize roots and leaves

**Fig. 2:**
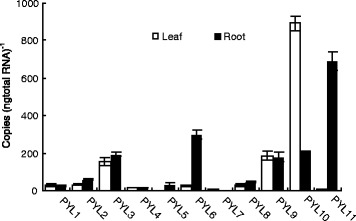
Absolute quantification of *ZmPYR/PYL/RCAR* transcripts in roots and leaves of maize seedlings under normal conditions. The top fully expanded leaves and primary roots of 15-day-old seedlings were sampled to extract total RNA. The absolute quantification of *ZmPYR/PYL/RCAR* was performed using serial dilution of plasmids carrying cDNAs of these genes. The results are the means of three biological replicates of four primary roots or leaves, each ± SE

Absolute quantification of the expression of *ZmPYR/PYL/RCAR* genes was performed by constructing a calibration curve using serial 10-fold dilutions of plasmids carrying *ZmPYR/PYL/RCAR* cDNA. The two most highly expressed genes, *ZmPYL10* and *ZmPYL11*, were present at 600–1000 copies per nanogram of total RNA (Fig. 2). *ZmPYL9, ZmPYL3* and *ZmPYL6* represented an intermediate group with copy numbers in the range of 200–400 copies per nanogram of total RNA, whereas *ZmPYL1*, *ZmPYL2*, *ZmPYL4*, *ZmPYL5, ZmPYL7* and *ZmPYL8* showed low expression, below 100 copies per nanogram of total RNA. Among the later group, *ZmPYL5* in the leaf and *ZmPYL7* in the root were very low to undetectable. The observed expression presents an organ-specific pattern for most of the moderate and highly expressed genes, with *ZmPYL11* and *ZmPYL6* primarily in roots and *ZmPYL10* in leaves. There was no difference between leaves and roots for *ZmPYL3 and ZmPYL9* gene expression, respectively*.*

### Dose- and time-dependent transcriptional responses of core ABA signaling components to ABA in maize roots

**Fig. 3:**
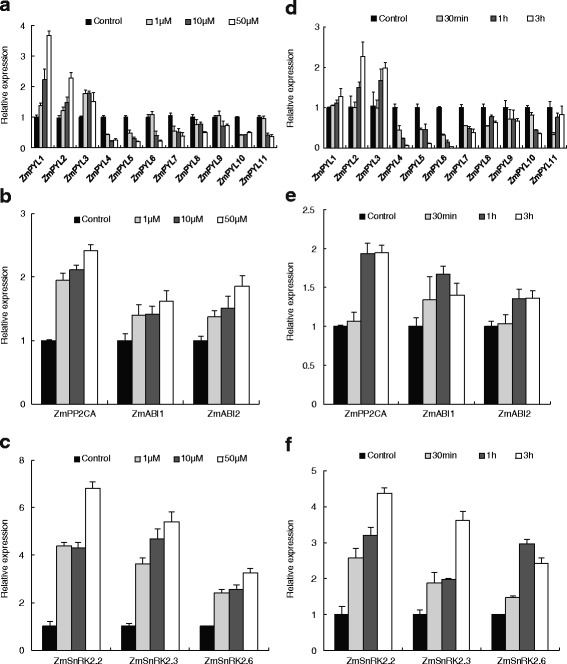
Transcriptional responses of ABA core signaling components to ABA, showing time- and dose-dependent patterns in maize primary roots. The root system of 15-day-old seedlings was exposed to 0 (control), 1, 10 or 50 μM ABA for 3 h (**a**, **b**, **c**) or to 1 μM ABA for 0 (control), 0.5, 1 or 3 h (**d**, **e**, **f**). Gene expression of ABA receptors *PYR/PYL/RCAR* (**a**, **d**), *PP2C* (**b**, **e**) and *SnRK2*s (**c**, **f**) was measured in the primary roots by real-time PCR. The results are the means of three biological replicates of four primary roots, each ± SE

Eleven ABA receptors (ZmPYL1-11), three type 2C protein phosphatases (ZmPP2CA, ZmABI1 and ZmABI2), and three protein kinases (ZmSnRK2.2, ZmSnRK2.3 and ZmSnRK2.6) were selected for further investigation as core ABA signaling components, and the time- and dose-dependent responses in expression of the corresponding genes to exogenous ABA were measured in maize primary roots. Figure 3 shows that the expression of these genes changed significantly after treatment with 1–50 μM ABA, with *ZmPYL4*-*11* being down-regulated in a dose-dependent manner and *ZmPYL1-3* up-regulated; *ZmPYL1* exhibited particularly high up-regulation. The levels of *ZmPP2C*s and *ZmSnRK2*s transcripts rose sharply in a dose-dependent manner following exposure to ABA.

Over the examined time gradient, the transcripts of *ZmPYL2* and *ZmPYL3* were dramatically increased after exposure to 1 μM ABA for 0.5 h, whereas the expression of genes *ZmPYL4-11* progressively decreased throughout the time-course. The expression patterns of PP2C family members were similar to each other, remaining stable in the initial 0.5 h, then slightly increasing and remaining stable thereafter. Kinase family transcripts increased continuously during the entire time-course of ABA treatment.

### Dose- and time-dependent transcriptional responses of core ABA signaling components to ABA in maize leaves

**Fig. 4:**
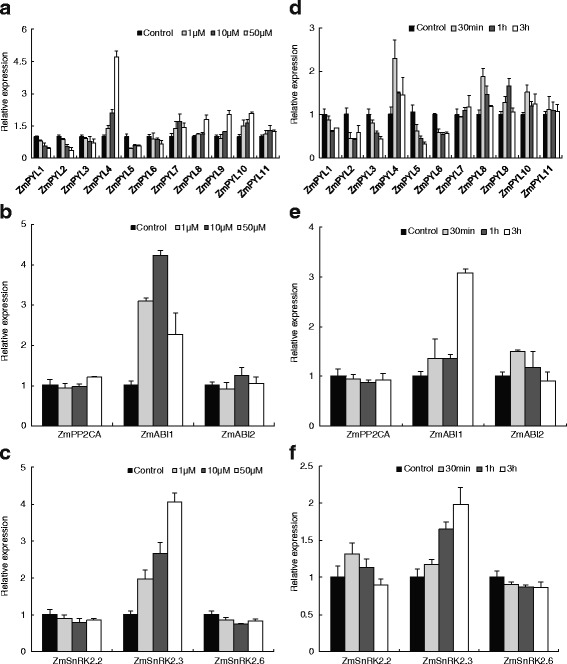
Transcriptional responses of ABA core signaling components to ABA, showing time- and dose-dependent patterns in maize leaves. The treatments were similar to those in Fig. 2. The topmost largest expanded leaves were sampled from the corresponding treatments. Gene expression of ABA receptors *PYR/PYL/RCAR* (**a**, **d**), *PP2C* (**b**, **e**) and *SnRK2*s (**c**, **f**) was measured by real-time PCR. The results are the means of three biological replicates of four leaves, each ± SE

The gene expression pattern of ABA receptors in maize leaves was entirely different from that in roots. Figure 4 shows that after treatment with 1–50 μM ABA, transcripts of *ZmPYL1* and *ZmPYL2,* which were up-regulated in roots, as well as that of *ZmPYL5* showed a continuously decreasing expression trend as the ABA concentration increased; in contrast, *ZmPYL7-10* transcripts increased under these conditions. The mRNA abundance of *ZmPYL3, ZmPYL6* and *ZmPYL11* remained stable. *ZmPYL4* gene expression was sharply induced by ABA in maize leaves, as observed for *ZmPYL1* in maize roots. Although the transcripts of *ZmABI1* and *ZmSnRK2.3* increased dramatically after ABA treatment, the transcripts of other members of their respective families remained stable or showed insignificant changes.

The transcripts of all of the examined ABA receptors presented a time-dependent response to ABA in maize leaves. Transcripts of *ZmPYL1-3* and *ZmPYL5-6* continuously decreased during 1 μM ABA treatment, whereas the transcripts of *ZmPYL4* and *ZmPYL8-10* increased after 0.5 h. The mRNA abundance of *ZmPYL7* and *ZmPYL11* remained stable during the time-course of 1 μM ABA treatment. The transcripts of *ZmABI1* and *ZmSnRK2.3* increased greatly, by 300 % and by 200 %, respectively. In contrast, the expression of genes *ZmPP2CA* and *ZmABI2* in the PP2C family and *ZmSnRK2.2* and *ZmSnRK2.6* in the SnRK2 family remained fairly stable from the beginning of treatment.

### Gene expression of core ABA signaling components in response to osmotic stress in maize roots

**Fig. 5:**
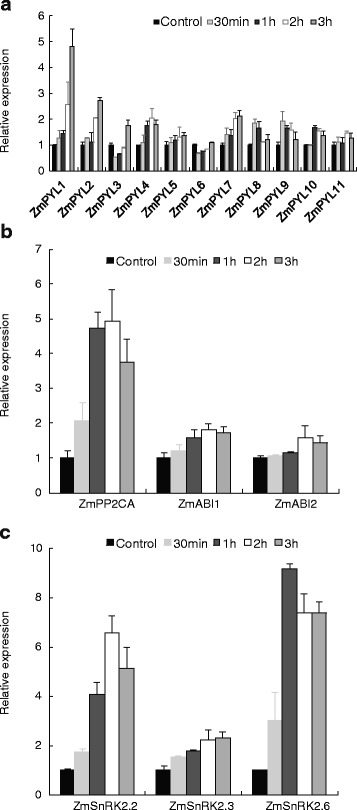
Transcriptional response of ZmPYLs (**a**), ZmPP2C (**b**) and ZmSnRK2 (**c**) to polyethylene glycol (PEG) in maize primary roots. The root system of 15-day-old seedlings was subjected to 20 % PEG for 0 (control), 0.5, 1, 2, or 3 h. Gene expression was measured by quantitative real-time PCR. The results are the means of three biological replicates of four primary roots, each ± SE

To compare the transcriptional responses of core ABA signaling components between ABA and osmotic stress, the maize root system was treated with a 20 % PEG solution. Figure 5 shows that with the exception of *ZmPYL1*, all of the remaining *ZmPYL* genes were activated by osmotic stress. The time-course of gene expression indicated that *ZmPYL1*, *ZmPYL4* and *ZmPYL7* are relatively early genes in the response to osmotic stress, whereas *ZmPYL3*, *ZmPYL5*, *ZmPYL8* and *ZmPYL10* are late-response genes. Interestingly, *ZmPYL1* was initially down-regulated (within 1 h), returning to the control level at 2 h and finally increasing above the control level. Both the *ZmPP2C* and *ZmSnRK2* gene families also exhibited a trend of up-regulated expression in response to 20 % PEG, especially the *ZmPP2CA*, *ZmSnRK2.2* and *ZmSnRK2.6* isoforms.

### Gene expression of core ABA signaling components in response to dehydration in maize leaves

**Fig. 6:**
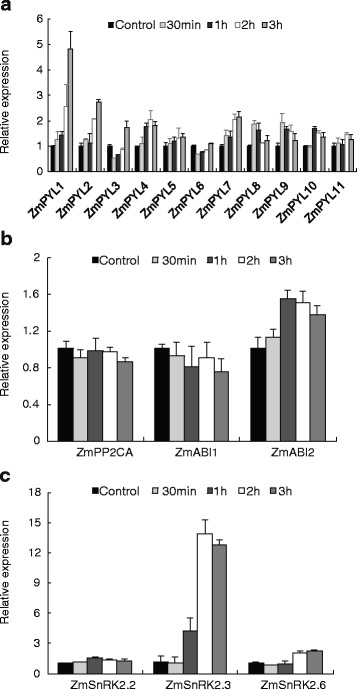
Transcriptional response of ZmPYLs (**a**), ZmPP2C (**b**) and ZmSnRK2 (**c**) to dehydration in leaves. The topmost largest expanded leaves of 15-day-old seedlings were detached for 0 (control), 0.5, 1, 2, or 3 h before analysis. Gene expression was measured by real-time PCR. The results are the means of three biological replicates of four leaves, each ± SE

To compare the transcriptional response of core ABA signaling components between ABA and dehydration stress, detached maize leaves were subjected to simulated dehydration stress. As shown in Fig. 6, ABA receptors *ZmPYL1-3*, *ZmPYL5-6* and *ZmPYL9-11* were down-regulated by dehydration but *ZmPYL4, ZmPYL7* and *ZmPYL8* significantly up-regulated. In addition, expression of *ZmABI2* increased sharply under dehydration, though *ZmPP2CA* and *ZmABI1* remained stable. Similarly, *ZmSnRK2.3* expression increased greatly under dehydration treatment, whereas the other two kinases, *ZmSnRK2.2* and *ZmSnRK2.6*, showed almost no response to dehydration.

## Discussion

It is well known that gene functions are closely related to the mechanism by which gene expression is regulated [3]. Although nearly all corresponding *ZmPYR/PYL/RCAR* genes are down-regulated by ABA in *Arabidopsis* [40], a portion of them were down-regulated and the others up-regulated in the crop plant maize. Overall, highly abundant genes in *Arabidopsis* show low expression in maize and vice versa under normal conditions. The present work addressed the species-specific mechanism regulating perception-related gene expression in response to ABA or osmotic/dehydration stress. The following sections mainly discuss the organ-, isoform-, stress type-, duration-, and intensity-specific regulation of the expression of core ABA signaling component genes.

### Organ-specific expression

Our findings clearly demonstrated that core ABA signaling component genes, especially PYR1/PYL/RCAR ABA receptor genes, in maize exhibit distinct expression patterns in response to abiotic stresses (or ABA) between roots and leaves, with isoforms that are up-regulated in roots being down-regulated in leaves, and vice versa. This is the first time that contrasting transcriptional responses of PYR1/PYL/RCAR ABA receptors to ABA have been observed between roots and leaves in a crop plant, and the results differ from those observed in the model plant *Arabidopsis* [25]. Organ-specific transcriptional regulation of signaling components may allow for variable responses to ABA between different parts of the plant under stress conditions. Our results also suggest that the relative contributions of individual PYR1/PYL/RCAR receptor genes to the stress response differ between roots and leaves. This type of distinguishable ABA sensitivity between leaves and roots may be due to the different water conditions experienced by these organs after plants are subjected to water deprivation-related stress [30].

Organ-specific expression patterns may also be related to the distinct redundant features of PYR1/PYL/RCAR receptor genes between roots and leaves. Although Antoni et al. [24] showed that PYL8 plays a nonredundant role in the regulation of root ABA sensitivity, this factor was necessary to generate a *pyr1pyl1pyl2pyl4* quadruple mutant (1124) to obtain robust ABA-insensitive phenotypes in shoots [7], and *pyr1pyl1pyl2pyl4pyl5pyl8* sextuple mutant 112458 is at least 1 order of magnitude more ABA insensitive than 1124 [25]. Recently, Zhao et al. [26] demonstrated that PYL8 promotes lateral root growth independent of the core ABA-SnRK2 signaling pathway. Using pyrabactin as an ABA mimic, we recently found that activation of PYR1 can significantly improve maize root hydraulic conductivity [41]. Therefore, the distinguishable sensitivity and gene redundancy observed between roots and leaves under both genetic modification of ABA receptors and ABA mimicry with synthetic ABA agonists should be given major attention. In practice, the former phenomenon can be addressed with an organ-specific promoter; for the latter, ABA agonists can be selectively used in either roots or shoots.

It is documented that the core SnRK2s involved in ABA signaling are not regulated by ABA treatment in *Arabidopsis* [42, 43]; however, the transcripts of SnRK2.2, SnRK2.3 and SnRK2.6 in maize roots and that of SnRK2.3 in maize leaves are significantly enhanced by ABA. The genes encoding the three types of PP2C phosphatases (ZmPP2CA, ZmABI1 and ZmABI2) examined in the present work all presented significant up-regulation patterns in maize roots, whereas only the transcript of ZmABI1 was induced by ABA in leaves. Thus, the transcriptional responses of both SnRK2s and PP2C to ABA are of an organ-specific nature.

### Isoform-specific expression

The present work revealed that each of the ABA receptors may play a distinct role in transmitting signals in maize, as indicated by their position in the phylogenetic tree of this receptor family. Based on phylogenetic analysis, ZmPYL1, ZmPYL2 and ZmPYL3, ZmPYL4, ZmPYL5, ZmPYL6 and ZmPYL7, and ZmPYL8, ZmPYL9, ZmPYL10 and ZmPYL11 share the same branches. As clearly shown by our expression profiling in maize roots, the transcripts of ZmPYL1-3 isoforms increased dramatically following ABA treatment, whereas those of the second subfamily members decreased depending on the ABA concentration. This distinct phenomenon was also observed in maize leaves, whereby *ZmPYL4* and *ZmPYL7-10* gene expression was up-regulated by ABA yet that of *ZmPYL1* and *ZmPYL2* was gradually down-regulated. Overall, the expression trends of *ZmPYL8-11*, in the third subfamily, were similar to those of the second subfamily. Transcripts of some members of the PYR/PYL/RCAR receptor family increased, whereas those of others declined, providing a potential mechanism for restoring ABA signaling homeostasis simultaneously in roots and leaves [44].

Following exposure to ABA, the level of *ZmPYL1* transcript increased dramatically in roots but that of *ZmPYL4* increased significantly in leaves. This may indicate that maize PYR1/PYL/RCAR ABA receptors that are homologous to dimeric-type *Arabidopsis* ABA receptors are mainly involved in transmitting ABA signaling in roots, whereas those that are homologous to monomeric-type *Arabidopsis* ABA receptors perform this function in leaves. However, this hypothesis is inconsistent with the results obtained by Antoni et al. [24] in *Arabidopsis*, indicating that the selective difference observed for PYR1/PYL/RCAR receptors between leaves and roots is not conserved among plant species. Overexpression of some monomeric, but not dimeric PYR/PYL receptors in both *Arabidopsis* and crop plants is known to enhance the response to ABA and plant drought resistance [3, 8, 14, 17, 45, 46], which may reflect differences in sensitivity to drought stress (or ABA) or different degrees of redundant and organ-specific gene expression (discussed in the above section). The root expression pattern of *PYL8* shows some specificity with respect to that of other PYR/PYL receptors in *Arabidopsis* [24], reflective of its crucial role in the hydrotropic response that occurs to guide root growth far from regions with low water potential. This further emphasizes the close correlation between gene expression and function. Isoform-specific gene expression of core components of ABA signaling is also observed under drought or dehydration conditions in other plant species [16, 18, 25, 47], and it has been shown that different subsets of phosphorylation events may depend on the functional presence of different subsets of the ABA receptor family for full ABA responsiveness [48]. Future studies will further elucidate details related to each PYR/PYL/RCAR receptor and their individual roles in the ABA-regulated response to osmotic stress.

### Stress duration (time)- and stress intensity (dose)-dependent responses

The time-dependent transcriptional response of ABA core signaling components to ABA or abiotic stress in maize indicated that some members tend to act as early-response genes, whereas others act as relatively late-response genes. In maize roots, *ZmPYL4-11* and *ZmSnRK2*s are relatively early genes in the response to ABA, whereas *ZmPYL1-3* and *ZmPP2C* are late genes; in response to osmotic stress, *ZmPYL8-9*, *ZmSnRK2*s and *ZmPP2CA* are early-response genes and *ZmPYL1-7, ZmPYL10-11, ZmABI1* and *ZmABI2* late-response genes. In maize leaves, *ZmPYL2*, *ZmPYL4*, *ZmPYL5*, *ZmPYL6*, *ZmPYL8-10* and *ZmABI2* are early genes in the response to ABA, and *ZmPYL1*, *ZmPYL3, ZmPYL7, ZmPYL11*, *ZmABI1* and *ZmSnRK2.3* are late-response genes. With the exception of *ZmPYL4*, which belongs to the late-response gene group together with *ZmPP2C* and *ZmSnRK2*, the other *ZmPYL* genes responded quickly to dehydration in maize leaves. Hence, we conclude that the time-dependent response of the core signaling components to ABA is not related to the phylogenetic results but is conserved between maize roots and leaves.

**Fig. 7:**
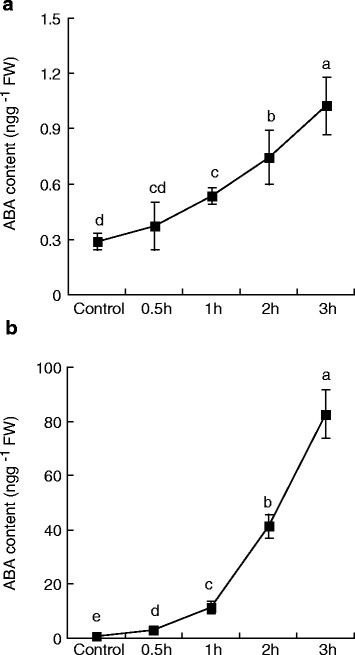
Endogenous ABA content of maize seedlings after stress. **a** Mean ABA content of maize roots after treatment with 20 % PEG6000 for 0 (control), 0.5, 1, 2, or 3 h. **b** The mean ABA content in maize leaves after detachment for 0 (control), 0.5, 1, 2, and 3 h. The results are the means of three biological replicates of five primary roots or leaves, each ± SE

All of the examined *ZmPYLs*, *ZmPP2C* and *ZmSnRK2* were sensitive to 1 μM ABA in maize roots under our experimental conditions; however, *ZmPYL7* and *ZmPYL11* in the receptor family, *ZmPP2CA* and *ZmABI2* in the *ZmPP2C* family, and *ZmSnRK2.2* and *ZmSnRK2.6* in the kinase family were not sensitive to this concentration of ABA in maize leaves. Our results are similar to the findings of Merilo et al. [19] in *Arabidopsis*, with the authors reporting that PYR/RCAR proteins appear to function in a dose-dependent manner in stomatal regulation induced by ozone, reduced air humidity, darkness and CO_2_. However, controlling the hormone dose/response ratio remains a challenge, as the hormone levels attained should be moderate to maintain a balance between their positive effects on stress tolerance and their negative effects on growth and development [1]. According to the model proposed by Cutler et al. [2], different stress durations (time) and intensities (dose) result in different ABA levels, and different PYR/PYL/RCAR members are therefore activated to sense changes in ABA in vivo. As the levels and temporal patterns of stress-derived endogenous ABA were different between the leaves and roots (Fig. 7), the perception mechanism may be distinct between these structures.

### Stress type-specific responses

Drought causes osmotic stress in organisms, and osmotic stress causes dehydration and inhibition of water uptake in plants. ABA accumulates under osmotic stress conditions and plays an important role in the stress response and tolerance of plants [49]. It has been shown that 25–50 % of the genes regulated by ABA are also modulated by drought or salinity. The present work also showed that the core signaling components present stress type-specific patterns of regulation. For example, in maize roots, the expression profile of *ZmPYL4* was different when the plant was exposed to ABA treatment and osmotic (PEG) stress. In leaves, dehydration and ABA treatments also induced distinct expression patterns for genes such as *ZmPYL5*, which may in turn lead to distinct functions in responses to different types of stresses.

As several biotic or abiotic stresses may occur simultaneously in field crops, several factors are involved in the accurate transcriptional regulation of ABA core signaling components in field-grown plants [48]. The sophisticated transcriptional regulation of the PYR/PYL/RCAR-mediated ABA signaling pathway and the different combinations of these signalosomes in vivo allows the plant to fine-tune its response to environmental fluctuations [49]. Moreover, we should not rule out ABA-independent mechanisms in response to osmotic-related abiotic stress. Indeed, the ratio of ABA-dependent/ABA-independent mechanisms in this case certainly affects ABA perception as well as the unique features of ABA core signaling components in the whole-plant response. Taken together, our results indicate that the core signaling components involved differs in the presence of exogenous ABA compared with stress-induced endogenous ABA in both leaves and roots.

In addition to the type of specificity indicated above, it should be noted that there are tissue-, cell-, subcellular- and even physiological process-specific responses by ABA core components [3, 7, 18, 50–52], and such variations in the expression and affinity of receptor and PP2C family members ultimately permit different responses over a wide range of ABA concentrations. It is possible that ABA perception at different levels may lead to different physiological outputs, further reflecting the sophisticated and diverse adaptive mechanisms that enable plants to survive under adverse environmental conditions. Additional studies are required to address the relationship between ABA core components and plant developmental stages, as stomatal sensitivity to ABA is acquired during leaf development through exposure to an increasingly dry atmosphere in the rosette plant *Arabidopsis* [30]. Remaining questions also include the roles of individual family members in specific abiotic stress responses or the integrated response to several types of stress.

## Conclusions

**Fig. 8:**
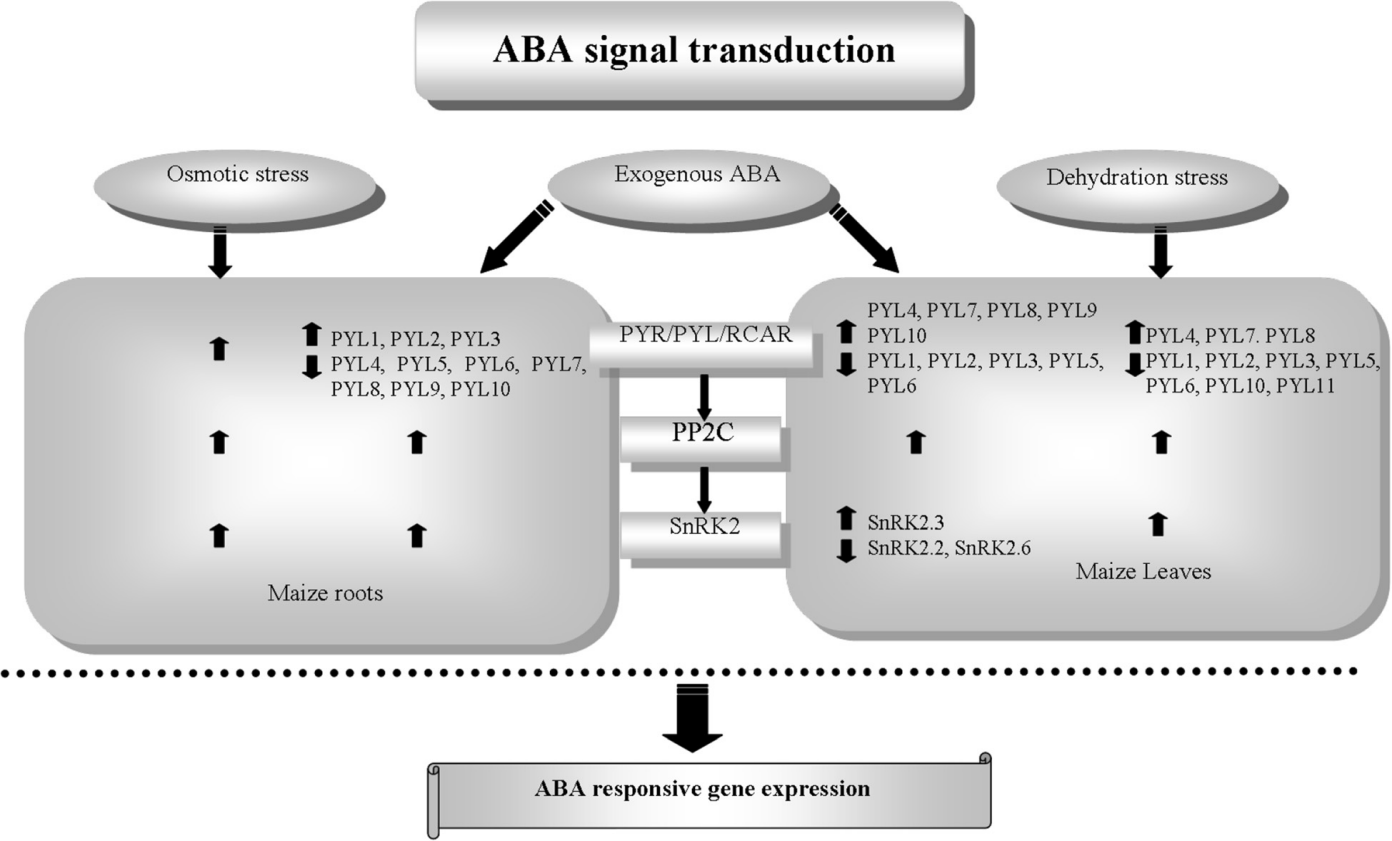
Contrasting transcriptional responses of core components of ABA signaling to exogenous ABA or osmotic stress-derived endogenous ABA between maize roots and leaves. The expression patterns of core ABA signaling components genes are illustrated based on real-time PCR results. The transcripts of *ZmPYL*s homologous to monomeric- and dimeric-type *Arabidopsis* ABA receptors were up- and down regulated by ABA or polyethylene glycol (PEG) in maize roots, respectively. This expression trend was reversed in leaves in the presence of ABA or dehydration. The transcripts of PP2Cs and SnRK2s were increased by ABA or osmotic stress in roots; in maize leaves, they were induced or maintained at constant levels, depending on the isoform within each family. Differences in core signaling components in the presence of exogenous ABA versus stress-induced endogenous ABA were observed in leaves and roots

In conclusion, the present study revealed the distinct expression profiles of 11 ABA receptors, 3 ZmPP2Cs and 3 ZmSnRK2s between maize roots and leaves in the presence of exogenous ABA or osmotic/dehydration-derived endogenous ABA (Fig. 8). Our results showed that transcripts of *ZmPYLs* that are homologous to dimeric-type *Arabidopsis* ABA receptors were up-regulated by ABA in maize primary roots, whereas those that are homologous to monomeric-type *Arabidopsis* ABA receptors were down-regulated. However, this trend was reversed in the leaves in the presence of ABA. Because *ZmPYL1* and *ZmPYL4* exhibit similar transcript abundance under normal conditions, our findings may represent a novel species-specific regulation mechanism of PYR/PYL/RCAR ABA receptor gene expression. This organ-specific ABA signaling plasticity endows the plant with plasticity in response to adverse environments, thus allowing acclimation to life on land. In addition to further elucidating the mechanism of PYR1/PYL modification at the transcriptional level, our results address the correlation between the function of ABA and its signal transduction mechanism.

## Methods

### Sequence information

Amino acid sequences of AtPYR/PYL/RCAR ABA receptors, AtPP2C protein phosphatases, and AtSnRK2 protein kinases of *Arabidopsis* were obtained from the protein database of National Center for Biotechnology Information (NCBI, http://www.ncbi.nlm.nih.gov/nucleotide) [6, 7]. mRNA sequences of ZmPYR/PYL/RCAR family genes reported by Hauser et al. [21] were obtained from Maize DB (http://www.maizesequence.org/index.html). ZmPP2C family members ZmHAB1, ZmPP2CA and ZmHAI were identified through *tblastn* searches, except for ZmABI1 and ZmABI2, which were characterized by Alexandrov et al. [35]. The ZmSnRK2 family was identified by Soderlund et al. [36]. These plant-specific serine/threonine kinases are divided into three subclasses in *Arabidopsis* [42, 43], among which SnRK2.2, SnRK2.3, and SnRK2.6, belonging to subclass III, have been found to be related to ABA signaling; these were selected as targets in the present study to examine their expression in maize seedlings under different stresses. Open reading frames (ORFs) were determined using NCBI ORF Finder (http://www.ncbi.nlm.nih.gov/gorf/orfig.cgi).

### Sequence alignment and phylogenetic analysis

The mRNA sequences of ZmPYLs, ZmPP2Cs, and ZmSnRK2s were aligned with their homologous sequences from *Arabidopsis,* as shown in Additional file 1: Figure S1, using ClustalX 2.1 software (http://www.clustal.org/clustal2/) and edited with BOXSHADE 3.21 (http://www.ch.embnet.org/software/BOX_form.html). Based on these alignments, phylogenetic trees were constructed according to the neighbor-joining (NJ) method using MEGA 5.1 software (http://www.megasoftware.net/), and the reliability of the various phylogenetic groups was evaluated through bootstrap analysis with 1000 replicates, as shown in Fig. 1.

### Plant materials

Seeds of maize (*Zea mays* L. cv. Zhengdan 958; Henan Academy of Agricultural Sciences, China) were germinated in a seed culture room at 25 °C. After germination, the maize seedlings were transferred to a controlled growth house and grown hydroponically in water for 4 days at 25 °C under a 16 h light/8 h dark cycle, with a light intensity at plant height was approximately 600 μmol m^−2^ s^−1^. Prior to treatments, the seedlings were further cultivated in 1/4 Hoagland nutrient solution for 11 days. The nutrient solutions were continuously aerated with an air pump and renewed at 3-day intervals.

### Exogenous ABA treatment

In the dose-dependent assay, 15-day-old maize seedlings were treated with 0 (control), 1, 10 or 50 μM of exogenous ABA ((±)ABA, Sigma) for 3 h. In the time-dependent assay, maize seedlings were exposed to 1 μM ABA for 0 (control), 0.5, 1 or 3 h. During treatment, stock solutions of ABA, which were prepared in ethanol, were added to the Hoagland nutrient solution of the same strength according to the indicated final concentration. After treatment, the top fully expanded leaves and primary roots were sampled, immediately frozen in liquid nitrogen, and subjected to RNA extraction. Four seedlings were used in each treatment, and the results are presented as the mean value of three biological replicates ± SE.

### Dehydration stress and osmotic stress treatments

Each stress treatment lasted for 0 (control), 0.5, 1, 2, or 3 h and was conducted using 15-day-old seedlings. The top fully expanded leaves were detached for dehydration treatment; for osmotic stress treatment, maize seedling roots were exposed to a 20 % PEG (−0.75 MPa) solution for different times, as indicated above. After treatment, all samples were immediately frozen in liquid nitrogen, powdered, mixed, and divided into two portions; one portion was used for real-time PCR analysis and the other for ABA determination. The samples were stored at−80 °C. Nine seedlings were used in each treatment, and the results are presented as the mean value of three biological replicates ± SE.

### RNA isolation and quantitative real-time PCR analysis

Total RNA from the samples was extracted using the TRIZOL reagent (TianGen, Beijing, China), and cDNA was synthesized through reverse transcription using the PrimeScript RT reagent kit (Takara, Dalian, China) according to the manufacturer’s instructions. Genomic DNA was eliminated using an RNase Free DNase I kit (Takara, Dalian, China), as suggested by the manufacturer.

The forward and reverse primers for each sequence used for real-time PCR were designed with Primer five software; the sequences are listed in Additional file 2: Table S1. All of the primer pairs were tested by PCR. A single product of the correct size for each gene was confirmed by agarose gel electrophoresis and double-strand sequencing (Invitrogen, Beijing, China). For absolute gene expression analysis, the amplified fragment of each gene was subcloned into the pMD18-T vector (Takara), and plasmids containing each specific gene were used in standard curve (log of cDNA dilution vs. C_t_) assays with serial 10-fold dilution. For the relative gene expression assay, the housekeeping gene alpha tubulin 6 (PCO104685b) was employed as an internal control, as it is assumed to exhibit uniform expression.

The real-time PCR procedure was established using the BioRad CFX96 system (America) with SYBR Premix Ex Taq (Takara, Dalian, China) according to the manufacturer’s recommendations. Each 20 μl reaction contained 10 μl of SYBR Premix Ex Taq mix, 0.5 μl of cDNA template (containing 100 ng of cDNA), 1.6 μl of primer mix (0.8 μl of each of the forward and reverse primers) and 7.9 μl of water. The following conditions for real-time PCR were designed and tested in a three-step assay: 95 °C/30 s (one cycle); 95 °C/5 s, 60 °C/30 s, 72 °C/30 s (40 cycles). The data were analyzed by the 2^–ΔΔCt^ method.

### ABA determination

ABA was measured as described by Shi et al. [53], with minor modifications. A 1.0 g powdered sample (fresh weight) was suspended in 8 ml of 80 % (v/v) methanol containing 200 mg L^−1^ of butylated hydroxytoluene and 500 mg L^−1^ of citric acid monohydrate on ice. The mixture remained stationary overnight at 4 °C before centrifugation for 15 min at 10,000 r/pm at 4 °C. The supernatant was subsequently collected, and the precipitate was extracted again for two h. The supernatants were then combined, dried under N_2_ and resuspended in 900 μL of 80 % methanol. After filtering the samples through a 0.45 μm filter, the ABA concentration in the extracts was analyzed using an LC-20AT high-performance liquid chromatography system (Shimadzu, Kinh Do, Japan) and an API 2000™ electrospray tandem mass spectrometer (Allen-Bradley, Milwaukee, WI, USA). (±)-ABA (A1049, Sigma) was used for the preparation of standard curves to quantify hormone concentrations in the samples.

### Availability of supporting data

The data sets supporting the results of this article are included within the article and its additional files.

## Additional files

Additional file 1: Figure S1. Multiple sequence alignment of *Arabidopsis thaliana* and *Zea mays* ABA core signaling components. (PDF 404 kb) Additional file 2: Table S1. Primers designed for the expression analysis of core ABA signaling component genes by quantitative RT-PCR (qRT-PCR). (PDF 7 kb)

## Abbreviations

ABA: abscisic acid
ABI: ABA-insensitive
CHLH/ABAR: Mg-chelatase H subunit/putative ABA receptor
GTG1/2: GPCR-type G proteins
NCBI: National Center for Biotechnology Information
NJ: neighbor-joining
ORFs: open reading frames
PEG: polyethylene glycol
PP2Cs: type 2C protein phosphatases
PYR1/PYL/RCAR: pyrabactin resistance 1/PYR1-like/regulatory components of ABA receptors
qRT-PCR: quantitative RT-PCR
SnRK2: sucrose nonfermenting 1-related subfamily 2

## References

[1] Peleg Z, Blumwald E (2011). Hormone balance and abiotic stress tolerance in crop plants. Curr Opin Plant Biol.

[2] Cutler SR, Rodriguez PL, Finkelstein RR, Abrams SR (2010). Abscisic acid: emergence of a core signaling network. Annu Rev Plant Physiol Plant Mol Biol.

[3] González-Guzmán M, Rodríguez L, Lorenzo-Orts L, Pons C, Sarrión-Perdigones A, Fernández MA (2014). Tomato PYR/PYL/RCAR abscisic acid receptors show high expression in root, differential sensitivity to the abscisic acid agonist quinabactin, and the capability to enhance plant drought resistance. J Exp Bot.

[4] Shen YY, Wang XF, Wu FQ, Du SY, Cao Z, Shang Y (2006). The Mg-chelatase H subunit is an abscisic acid receptor. Nature.

[5] Pandey S, Nelson DC, Assmann SM (2009). Two novel GPCR-type G proteins are abscisic acid receptors in Arabidopsis. Cell.

[6] Ma Y, Szostkiewicz I, Korte A, Moes D, Yang Y, Christmann A (2009). Regulators of PP2C phosphatase activity function as abscisic acid sensors. Science.

[7] Park SY, Fung P, Nishimura N, Jensen DR, Fujii H, Zhao Y (2009). Abscisic acid inhibits PP2Cs via the PYR/PYL family of ABA-binding START proteins. Science.

[8] Santiago J, Rodrigues A, Saez A, Rubio S, Antoni R, Dupeux F (2009). Modulation of drought resistance by the abscisic acid receptor PYL5 through inhibition of clade A PP2Cs. Plant J.

[9] Nishimura N, Sarkeshik A, Nito K, Park SY, Wang A, Carvalho PC (2010). PYR/PYL/RCAR family members are major in‐vivo ABI1 protein phosphatase 2C‐interacting proteins in Arabidopsis. Plant J.

[10] Fujii H, Chinnusamy V, Rodrigues A, Rubio S, Antoni R, Park SY (2009). In vitro reconstitution of an abscisic acid signalling pathway. Nature.

[11] Umezawa T, Nakashima K, Miyakawa T, Kuromori T, Tanokura M, Shinozaki K (2010). Molecular basis of the core regulatory network in ABA responses: sensing, signaling and transport. Plant Cell Physiol.

[12] Umezawa T, Sugiyama N, Mizoguchi M, Hayashi S, Myouga F, Yamaguchi-Shinozaki K (2009). Type 2C protein phosphatases directly regulate abscisic acid-activated protein kinases in Arabidopsis. Proc Natl Acad Sci U S A.

[13] Zhao Y, Chan Z, Gao J, Xing L, Cao M, Yu C (2016). ABA receptor PYL9 promotes drought resistance and leaf senescence. Proc Natl Acad Sci U S A.

[14] Saavedra X, Modrego A, Rodríguez D, González-García MP, Sanz L, Nicolás G (2010). The nuclear interactor PYL8/RCAR3 of *Fagus sylvatica* FsPP2C1 is a positive regulator of abscisic acid signaling in seeds and stress. Plant Physiol.

[15] Chai YM, Jia HF, Li CL, Dong QH, Shen YY (2011). FaPYR1 is involved in strawberry fruit ripening. J Exp Bot.

[16] Sun L, Wang YP, Chen P, Ren J, Ji K, Li Q (2011). Transcriptional regulation of SlPYL, SlPP2C, and SlSnRK2 gene families encoding ABA signal core components during tomato fruit development and drought stress. J Exp Bot.

[17] Kim H, Hwang H, Hong JW, Lee YN, Ahn IP, Yoon IS (2012). A rice orthologue of the ABA receptor, OsPYL/RCAR5, is a positive regulator of the ABA signal transduction pathway in seed germination and early seedling growth. J Exp Bot.

[18] Romero P, Lafuente MT, Rodrigo MJ (2012). The Citrus ABA signalosome: identification and transcriptional regulation during sweet orange fruit ripening and leaf dehydration. J Exp Bot.

[19] Merilo E, Laanemets K, Hu H, Xue S, Jakobson L, Tulva I (2013). PYR/RCAR receptors contribute to ozone-, reduced air humidity-, darkness-and CO_2_-induced stomatal regulation. Plant Physiol.

[20] Klingler JP, Batelli G, Zhu JK (2010). ABA receptors: the START of a new paradigm in phytohormone signalling. J Exp Bot.

[21] Hauser F, Waadt R, Schroeder JI (2011). Evolution of abscisic acid synthesis and signaling mechanisms. Curr Biol.

[22] Komatsu K, Suzuki N, Kuwamura M, Nishikawa Y, Nakatani M, Ohtawa H (2013). Group A PP2Cs evolved in land plants as key regulators of intrinsic desiccation tolerance. Nature Commu.

[23] Sharp RE, LeNoble ME (2002). ABA, ethylene and the control of shoot and root growth under water stress. J Exp Bot.

[24] Antoni R, Gonzalez-Guzman M, Rodriguez L, Peirats-Llobet M, Pizzio GA, Fernandez MA (2013). PYRABACTIN RESISTANCE1-LIKE8 plays an important role for the regulation of abscisic acid signaling in root. Plant Physiol.

[25] Gonzalez-Guzman M, Pizzio GA, Antoni R, Vera-Sirera F, Merilo E, Bassel GW (2012). Arabidopsis PYR/PYL/RCAR receptors play a major role in quantitative regulation of stomatal aperture and transcriptional response to abscisic acid. Plant Cell.

[26] Zhao Y, Xing L, Wang X, Hou YJ, Gao J, Wang P (2014). The ABA receptor PYL8 promotes lateral root growth by enhancing MYB77-dependent transcription of auxin-responsive genes. Sci Signal.

[27] Yamaguchi-Shinozaki K, Shinozaki K (2006). Transcriptional regulatory networks in cellular responses and tolerance to dehydration and cold stresses. Annu Rev Plant Physiol Plant Mol Biol.

[28] Virlouvet L, Ding Y, Fujii H, Avramova Z, Fromm M (2014). ABA signaling is necessary but not sufficient for RD29B transcriptional memory during successive dehydration stresses in *Arabidopsis thaliana*. Plant J.

[29] Ribeiro DM, Desikan R, Bright J, Confraria A, Harrison J, Hancock JT (2009). Differential requirement for NO during ABA‐induced stomatal closure in turgid and wilted leaves. Plant Cell Environ.

[30] Pantin F, Renaud J, Barbier F, Vavasseur A, Le Thiec D, Rose C (2013). Developmental priming of stomatal sensitivity to abscisic acid by leaf microclimate. Curr Biol.

[31] Wang YG, Yu HQ, Zhang YY, Lai CX, She YH, Li WC (2014). Interaction between abscisic acid receptor PYL3 and protein phosphatase type 2C in response to ABA signaling in maize. Gene.

[32] Broz AK, Thelen JJ, Muszynski MG, Miernyk JA, Randall DD (2001). ZMPP2, a novel type‐2C protein phosphatase from maize. J Exp Bot.

[33] Wei K, Pan S (2014). Maize protein phosphatase gene family: identification and molecular characterization. BMC Genomics.

[34] Huai J, Wang M, He J, Zheng J, Dong Z, Lv H (2008). Cloning and characterization of the SnRK2 gene family from Zea mays. Plant Cell Rep.

[35] Ying S, Zhang DF, Li HY, Liu YH, Shi YS, Song YC (2011). Cloning and characterization of a maize SnRK2 protein kinase gene confers enhanced salt tolerance in transgenic Arabidopsis. Plant Cell Rep.

[36] Wei K, Wang Y, Xie D (2014). Identification and expression profile analysis of the protein kinase gene superfamily in maize development. Mol Breeding.

[37] Wei K, Wang Y, Zhong X, Pan S (2014). Protein kinase structure, expression and regulation in maize drought signaling. Mol Breeding.

[38] Alexandrov NN, Brover VV, Freidin S, Troukhan ME, Tatarinova TV, Zhang H (2009). Insights into corn genes derived from large-scale cDNA sequencing. Plant Mol Biol.

[39] Soderlund C, Descour A, Kudrna D, Bomhoff M, Boyd L, Currie J (2009). Sequencing, mapping, and analysis of 27,455 maize full-length cDNAs. PLoS Genet.

[40] Boursiac Y, Léran S, Corratgé-Faillie C, Gojon A, Krouk G, Lacombe B (2013). ABA transport and transporters. Trends Plant Sci.

[41] Fan W, Li J, Jia J, Wang F, Cao C, Hu J (2015). Pyrabactin regulates root hydraulic properties in maize seedlings by affecting PIP aquaporins in a phosphorylation-dependent manner. Plant Physiol Biochem.

[42] Fujii H, Zhu JK (2009). Arabidopsis mutant deficient in 3 abscisic acid-activated protein kinases reveals critical roles in growth, reproduction, and stress. Proc Natl Acad Sci U S A.

[43] Fujita Y, Nakashima K, Yoshida T, Katagiri T, Kidokoro S, Kanamori N (2009). Three SnRK2 protein kinases are the main positive regulators of abscisic acid signaling in response to water stress in Arabidopsis. Plant Cell Physiol.

[44] Finkelstein R (2013). Abscisic acid synthesis and response. Arabidopsis Book.

[45] Pizzio GA, Rodriguez L, Antoni R, Gonzalez-Guzman M, Yunta C, Merilo E (2013). The PYL4 A194T mutant uncovers a key role of PYR1-LIKE4/PROTEIN PHOSPHATASE 2CA interaction for abscisic acid signaling and plant drought resistance. Plant Physiol.

[46] Kim H, Lee K, Hwang H, Bhatnagar N, Kim DY, Yoon IS (2014). Overexpression of PYL5 in rice enhances drought tolerance, inhibits growth, and modulates gene expression. J Exp Bot.

[47] Wang Y, Wu Y, Duan C, Chen P, Li Q, Dai S (2012). The expression profiling of the CsPYL, CsPP2C and CsSnRK2 gene families during fruit development and drought stress in cucumber. J Plant Physiol.

[48] Ramegowda V, Senthil-Kumar M (2015). The interactive effects of simultaneous biotic and abiotic stresses on plants: mechanistic understanding from drought and pathogen combination. J Plant Physiol.

[49] Fujita Y, Fujita M, Shinozaki K, Yamaguchi-Shinozaki K (2011). ABA-mediated transcriptional regulation in response to osmotic stress in plants. J Plant Res.

[50] Szostkiewicz I, Richter K, Kepka M, Demmel S, Ma Y, Korte A (2010). Closely related receptor complexes differ in their ABA selectivity and sensitivity. Plant J.

[51] Xu ZY, Kim DH, Hwang I (2013). ABA homeostasis and signaling involving multiple subcellular compartments and multiple receptors. Plant Cell Rep.

[52] Yin Y, Adachi Y, Ye W, Hayashi M, Nakamura Y, Kinoshita T (2013). Difference in abscisic acid perception mechanisms between closure induction and opening inhibition of stomata. Plant Physiol.

[53] Shi WG, Li H, Liu TX, Polle A, Peng CH, Luo ZB (2015). Exogenous abscisic acid alleviates zinc uptake and accumulation in Populus × canescens exposed to excess zinc. Plant Cell Environ.

