# Correction to: Contrasting transcriptional responses of PYR1/PYL/RCAR ABA receptors to ABA or dehydration stress between maize seedling leaves and roots

**DOI:** 10.1186/s12870-018-1283-8

**Published:** 2018-04-30

**Authors:** Wenqiang Fan, Mengyao Zhao, Suxin Li, Xue Bai, Jia Li, Haowei Meng, Zixin Mu

**Affiliations:** College of Life Sciences, Northwest A&F University, Yangling, 712100 Shaanxi China

## Correction

Following publication of the original article [1], a reader spotted that the article appears to have some misplaced/duplicated figures. In particular, Fig. 5a and Fig. 6a appear to be identical, and do not match what is written in the text. The authors apologized for this oversight and supplied the original pictures, which are reproduced below.

**Fig. 5:**
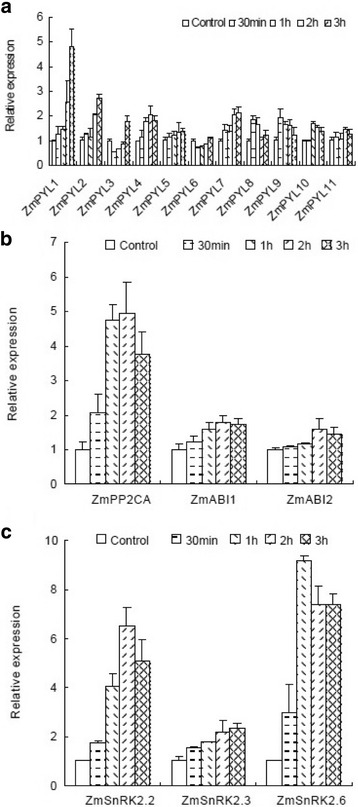
Transcriptional response of ZmPYLs (**a**), ZmPP2C (**b**) and ZmSnRK2 (**c**) to polyethylene glycol (PEG) in maize primary roots. The root system of 15-day-old seedlings was subjected to 20% PEG for 0 (control), 0.5, 1, 2, or 3 h. Gene expression was measured by quantitative real-time PCR. The results are the means of three biological replicates of four primary roots, each ± SE

**Fig. 6:**
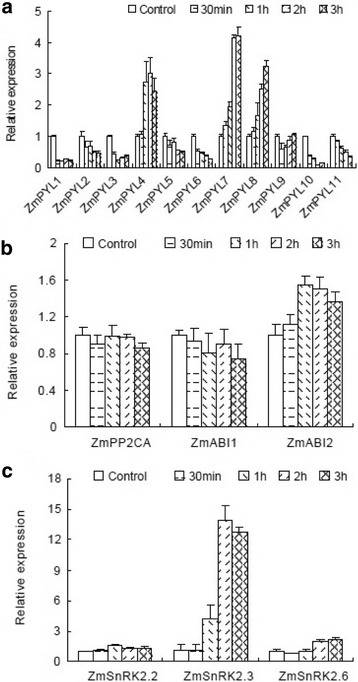
Transcriptional response of ZmPYLs (**a**), ZmPP2C (**b**) and ZmSnRK2 (**c**) to dehydration in leaves. The topmost largest expanded leaves of 15-day-old seedlings were detached for 0 (control), 0.5, 1, 2, or 3 h before analysis. Gene expression was measured by real-time PCR. The results are the means of three biological replicates of four leaves, each ± SE
