# Supplementary material for: Contrasting transcriptional responses of PYR1/PYL/RCAR ABA receptors to ABA or dehydration stress between maize seedling leaves and roots
Source: BMC Plant Biol. 2016 Apr 21;16:99. doi: 10.1186/s12870-016-0764-x (PMC4839062; doi:10.1186/s12870-016-0764-x)

Figure S1. Multiple sequence alignment of the *Arabidopsis thaliana* and *Citrus sinensis* ABA signalling core components. (A) START-like domain of the ABA-receptors, delimited by black triangles below the alignment. ABA-binding sites are indicated by arrows and PP2Cs interaction sites by asterisks. Boxes indicate *Gate* and *Latch* loops. (B) PP2C-like domain of the clade-A PP2Cs proteins, delimited by black triangles below the alignment. Metal-binding sites are pointed out by asterisks and phosphatase activity regulatory sequences are underlined. (C) Subclass III SnRK2s full protein sequences. Black triangles below the alignment delimit protein kinase domain, asterisk indicate ATP binding site and plus symbol highlights the proton acceptor active site.

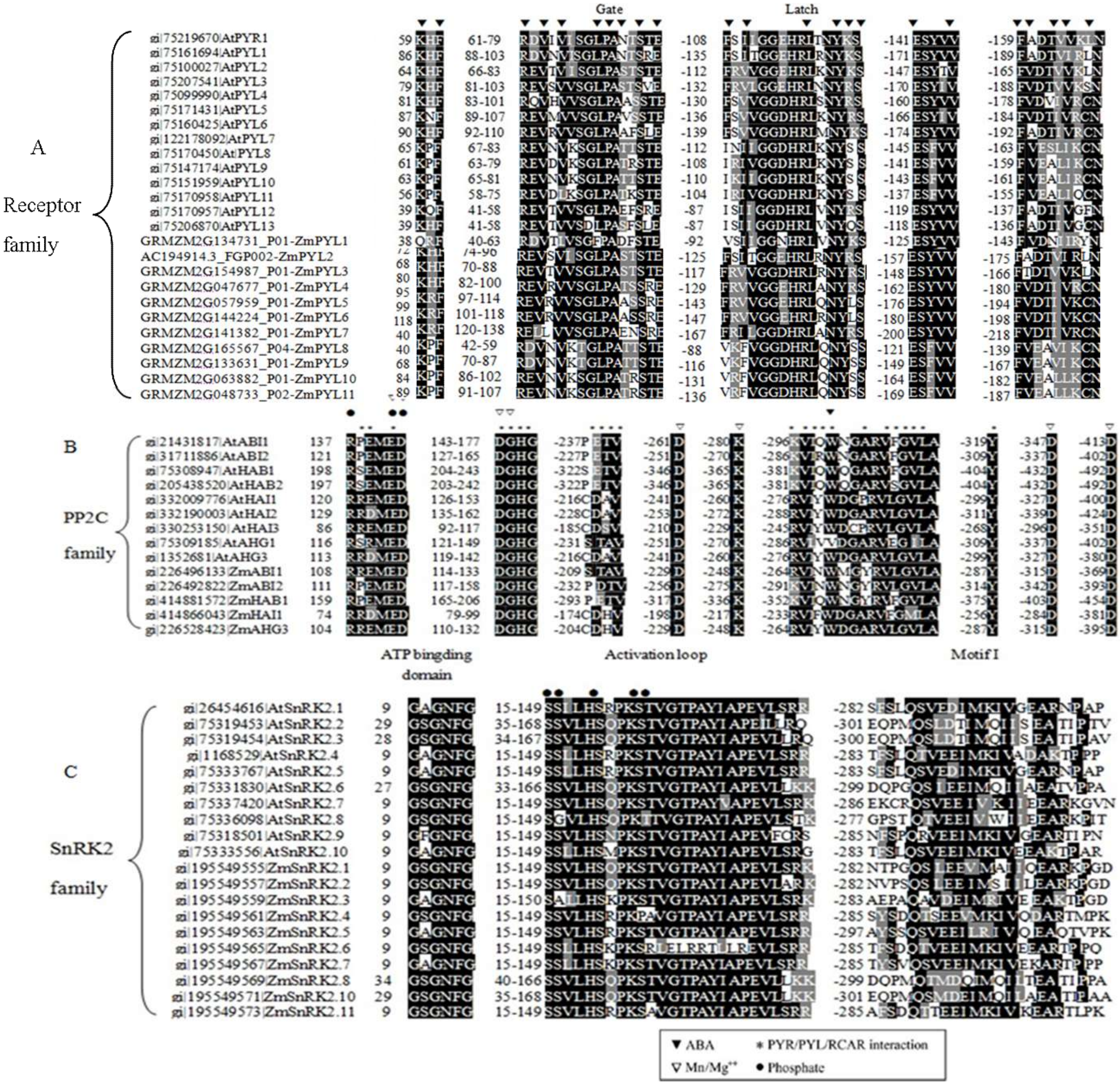

Supplement: Additional file 1: Figure S1. — Multiple sequence alignment of Arabidopsis thaliana and Zea mays ABA core signaling components. (PDF 404 kb) [file 12870_2016_764_MOESM1_ESM.pdf]
