# Supplementary material for: Contrasting transcriptional responses of PYR1/PYL/RCAR ABA receptors to ABA or dehydration stress between maize seedling leaves and roots
Source: BMC Plant Biol. 2016 Apr 21;16:99. doi: 10.1186/s12870-016-0764-x (PMC4839062; doi:10.1186/s12870-016-0764-x)
Supplement: Additional file 2: Table S1. — Primers designed for the expression analysis of core ABA signaling component genes by quantitative RT-PCR (qRT-PCR). (PDF 7 kb) [file 12870_2016_764_MOESM2_ESM.pdf]

Table S1 Primers designed for gene expression analyses by quantitative RT-PCR (qRT-PCR).

Gene	Forward Primer	Reverse Primer	Accession
ZmPYL1	GGAGAAACGCAGCAGCAA	GTCCAGTCCAAATCCATCCAC	NM_001174992.1
ZmPYL2	AGGGTCTTCGGCTTCTCCATC	GGTGTCGTCCTCGGTGTTGC	NM_001157214.1
ZmPYL3	GACATCGTCGTCAAGCTCAACCT	GGCAATTTGCAGTAGCAACGG	NM_001176567.1
ZmPYL4	GTTTGCGACACAGAGTACAAATCA	GCAGGAACACACACAGCACA	NM_001143005.2
ZmPYL5	GCACCGGCTCCAGAACTA	CACGCAGTCACGCCTACA	NM_001154788.1
ZmPYL6	CCATCGTCAAGTGCAACCT	GGGGACACTAATGTGAAAAGG	NM_001319727.1
ZmPYL7	CCGAACCACACAGTAACAATAGATG	AGGAGAGGTGGAGATTGGGTTAG	EU964115.1
ZmPYL8	TCCAGAACTACTCATCCATCATAAC	ATGTCTCGTCCTTCGTGTTG	BT055711.1
ZmPYL9	GAAAGAGGAGCAGACAAACAAAATC	GACGGATGAGGACGAGGAAA	NM_001147497.1
ZmPYL10	GGCAACACCAAGGACGAGA	CAGTAGCAGTAGCAGTCCCTCAAA	EU959289.1
ZmPYL11	ACCTCGTCGTCTGCTTTTCC	TCCATCTCCACCACCATCAC	EU967268.1
ZmPP2CA	GATGCTGGCTCAACTGCCT	GAAACCGCAATACCCTGTCC	NM_001155565.2
ZmPP2C ABI1	CACATCGTGGTCGCCAACT	CCACCTGCTGATTCTACCCTTT	NM_001158117.1
ZmPP2C ABI2	AGCACTGGGAGAAGGTCTTTGT	GGCAACGATGACATGCGAT	NM_001156830.1
ZmSnRK2.2	CGAAGAATTTCCGCAAGACG	TCGCCACAAAGATCCGAGAC	NM_001137717.2
ZmSnRK2.3	CGCCTTCTGTCTCAGATATTCG	GCATCCGCGTCCTTGTAGTT	NM_001143024.1
ZmSnRK2.6	ACATCGGTTCCGGCAACTT	TCCCTCGCCACATTCTCGT	NM_001143324.1
